# Anxiety Changes Depersonalization and Derealization Symptoms in Vestibular Patients

**DOI:** 10.1155/2014/847054

**Published:** 2014-01-28

**Authors:** Ognyan I. Kolev, Spaska O. Georgieva-Zhostova, Alain Berthoz

**Affiliations:** ^1^University Hospital of Neurology and Psychiatry “St. Naum”, 1113 Sofia, Bulgaria; ^2^Laboratoire de Physiologie de la Perception et de l'Action, CNRS and Collège de France, 75231 Paris, France

## Abstract

*Background.* Depersonalization and derealization are common symptoms reported in the general population. *Objective.* The aim of the present study was to establish the relationship between anxiety and depersonalization and derealization symptoms in patients with peripheral vestibular disorders. *Methods.* Twenty-four vestibular patients with anxiety and 18 vestibular patients without anxiety were examined for depersonalization and derealization symptoms. They were also compared to healthy controls. *Results.* The results revealed that anxiety consistently changes depersonalization and derealization symptoms in vestibular patients. They are more frequent, more severe, and qualitatively different in vestibular patients with anxiety than in those without anxiety. *Conclusion.* Anxiety has an effect on depersonalization and derealization symptoms in vestibular patients. The various hypotheses about the underlying mechanism of this effect were discussed.

## 1. Introduction

Depersonalization (Dp) is an alteration in the perception or experience of the self which results in a feeling of being detached, as if one is an external observer of one's mental processes or body. Derealization (Dr) is an experience of the external world that appears strange or unreal [1]. Dp/Dr symptoms are common in the general population [2–4]. Abnormal vestibular stimulation with calorics has been found to provoke feelings of unreality in healthy subjects [5–7]. Our earlier studies also showed different unreal perceptions of self-motion, perceived unequally by the different parts of the body [8]; moreover, we discovered vestibularly evoked visual hallucinations [9]. All this indicates the multisensory effects of vestibular stimulation. Sang et al. have established that patients with peripheral vestibular disease often report symptoms of Dp/Dr [10]. They proposed that derealization occurs in these patients because their distorted vestibular signals create a misleading frame of spatial reference, which does not match with the other senses, giving rise to illusory, “unreal” perceptions of the patient's transactions in the physical world. During the acute phase of a unilateral peripheral vestibular lesion the poor spatial orientation of vestibular patients cooccurs with Dp/Dr symptoms, including attention/concentration difficulties and somatic depression symptoms. Months later Dp/Dr symptoms in these patients decrease, but somatic symptoms of depression persist [11]. In addition, those vestibular patients who have an acquired deficiency of other special senses, for example, vision and hearing, also have more frequent and severe Dp/Dr symptoms than do healthy controls. These symptoms are always associated with symptoms of common mental disorders [12].

The nature and localization of brain dysfunction associated with a depersonalization disorder have not yet been conclusively clarified. The results of a functional imaging study of patients with depersonalization disorder suggest that abnormalities occur primarily along sequential hierarchical areas (unimodal and crossmodal) of the visual, somatosensory, and auditory processing pathways, as well as in areas responsible for the integrated body schema (specifically area 7B). This is consistent with the proposal that the inferior parietal cortex is concerned with spatial orientation as well as visuomotor and vestibular function [13]. Kahane et al. [14] showed by electrical stimulation in epileptic patients, a procedure initially proposed by Penfield, that the vestibular cortex at the temporoparietal junction is involved exactly in “body awareness.” In addition, they showed that a large area, the peri-sylvian vestibular cortex, is involved in spatial orientation. Dp/Dr symptoms such as unambiguous self-location, egocentric visuospatial prospective, and out-of-body experience were suggested to be related to neural activity at the temporoparietal junction [15] in epilepsy. They tend to occur if there is coexisting vestibular dysfunction [16]. Phenomenological similarities between visual hypoemotionality and derealization suggest that the underlying mechanism may be a disruption of the process by means of which perception becomes emotionally colored. Phenomenological overlaps with asomatognosia suggest that depersonalization might result from parietal mechanisms that impair the experience of body ownership and agency [17]. Another aspect of the Dp/Dr is that these symptoms are very common in people with anxiety. Subjects experiencing depersonalization and derealization report more anxiety [18]. Mood, anxiety, and personality disorders are often comorbid with depersonalization disorders [19, 20]. On the basis of the idea that anxiety and depersonalization are intimately related, Hunter et al. [21] recently proposed a cognitive-behavioral model of depersonalization. Patients with persisting vestibular symptoms had persisting anxiety symptoms [22–25].

Our aim in the present study was to establish the relationship between anxiety and depersonalization and derealization symptoms in patients with peripheral vestibular disorders. We posed the question as to whether anxiety changes qualitatively and/or quantitatively Dp/Dr symptoms in these patients.

## 2. Subjects and Methods

Forty-two patients with peripheral vestibular disease (35 females and 7 males; mean age 42 (SD ± 10.62) years, range 27–65 years) and 18 healthy age-matched control subjects voluntarily participated. They were not compensated for participating. Subjects younger or older than this age range were excluded from the study. The subjects gave their written informed consent to take part in the study, which was approved by the Ethics Committee of Medical University, Sofia. General characteristics of the subjects are shown in Table 1.

All subjects were examined at the Department of Neurology and Neurotology at the University Hospital “St. Naum,” Sofia. The 18 healthy controls were selected from the hospital staff or were recruited by public announcement. The 12 females and 6 males were screened to ensure that they had never been diagnosed to have neurological or vestibular dysfunction, hearing loss, or dizziness during the past year. They were also not under psychiatric care or on psychotropic medication. None had strabismus or ophthalmologic disorders other than corrected refractive errors.

The vestibular group consisted of inpatients at the “Saint Naum” hospital. All had a clinical diagnosis of a vestibular disorder based on the patient's history, detailed neurological and neurootological examinations, eye movement examination, hearing, posturography, positional maneuvers, rotation and caloric testing (30° and 44°C) (equipment of Synapsys Inc., USA) during their hospitalization.

Clinical diagnoses are listed in Table 2.

All patients had complaints of dizziness and imbalance and denied a history of other neurological or psychiatric disorders. Hearing was normal in 35 patients; 3 patients had mild to moderate, high-frequency, bilateral hearing loss and 1 had moderate to severe bilateral hearing loss, all frequencies; 3 patients had moderate to severe high-frequency unilateral hearing loss. Hearing loss was concomitant with vestibular disease or due to presbyacusis. None had strabismus or ophthalmologic disorders. Ten patients had corrected refractive errors.

All subjects completed two written tests at the outset.

(1) Hospital Anxiety and Depression Scale (HADS)—a 14-item self-reported instrument designed to screen for the presence and severity of symptoms of depression and anxiety over the past week. It is a brief and useful screening tool for symptoms of depression and anxiety. The General Health Questionnaire (GHQ) has been used for this kind of study before [10, 12]. The HADS test was chosen instead of the GHQ because several articles [26, 27] had indicated its sensitivity to change and better performance in all analyses. The items in HADS are scored on a 0–3 scale: HADS-D (depression) and HADS-A (anxiety) subscale scores (range 0–21) are derived by adding the seven items on each scale. For both subscales, scores in the range of 0–7 are considered normal; 8–10; mild and 11–14 are moderate; are 15–21 are severe.

According to the results of the HAD-A subscale the subjects were divided into three groups:

- 18 healthy controls;
- 18 patients with peripheral vestibular disorder without anxiety symptoms—10 had acute peripheral vestibular dysfunction and 8 had had complaints of dizziness or imbalance for more than 1 year.
- 24 patients with peripheral vestibular disorder and anxiety symptoms—11 patients had acute peripheral vestibular dysfunction and 13 had had a vestibular disorder for more than 1 year.

The anxiety was confirmed by a psychiatrist.

(2) The 28-item depersonalization/derealization inventory by Cox and Swinson grades the severity of each item on a five-point scale, where 0 = does not occur, 1 = mild, 2 = moderate, 3 = severe, and 4 = very severe. Healthy subjects were instructed to fill in the answers according to their life experience. The vestibular patients were asked the following question: “Since the first time you had vertigo, have you ever had these types of experiences?”

*Data Processing and Analysis.* The score for the HADS was obtained from the two subscales for anxiety and depression on 0–3 scale. The score for the Dp/Dr inventory was calculated as the sum of the individual scores of each of the 28 items. The statistical analyses were performed with Statistica 7.0 (Stat Soft Inc., USA, 2004), and statistical significance was set at *P* < 0.05. A descriptive statistic of demographic data and clinical variables was applied. The Spearman's correlation coefficients, Mann-Whitney *U* test (for continuous variables), and the Fisher's exact test (for categorical variables) were used to examine the significant differences among the groups. Multivariate analysis was performed by using the linear regression model. Each independent factor that was statistically significant at the bivariate level (*P* < 0.1) was included in the analysis. Discriminant function analysis was used to identify the items that could discriminate between vestibular patients with and without anxiety at a significant level (*P* < 0.05).

## 3. Results

### 3.1. Healthy Subjects

**Figure 1:**
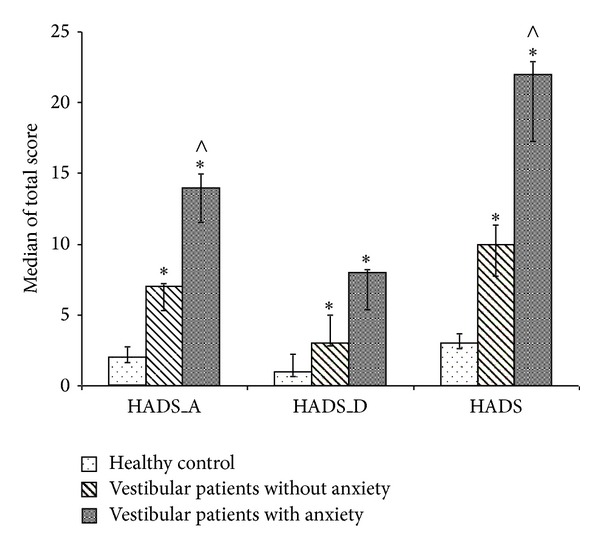
Median values and 95% confidence interval for HADS-A, HADS-D, and HADS scores for healthy subjects, vestibular patients without anxiety, and vestibular patients with anxiety. *Significance between healthy subjects and vestibular patients, *P* < 0.05 and ^∧^significance between both vestibular patients groups, *P* < 0.05 (Mann-Whitney *U* test).

Healthy subjects had a HADS total score ranging from 0 to 8 (median 3). The subscales for anxiety (HADS-A) ranged from 0 to 4 (median 2) and for depression (HADS-D) ranged from 0 to 4 (median 1) (Figure 1).

**Figure 2:**
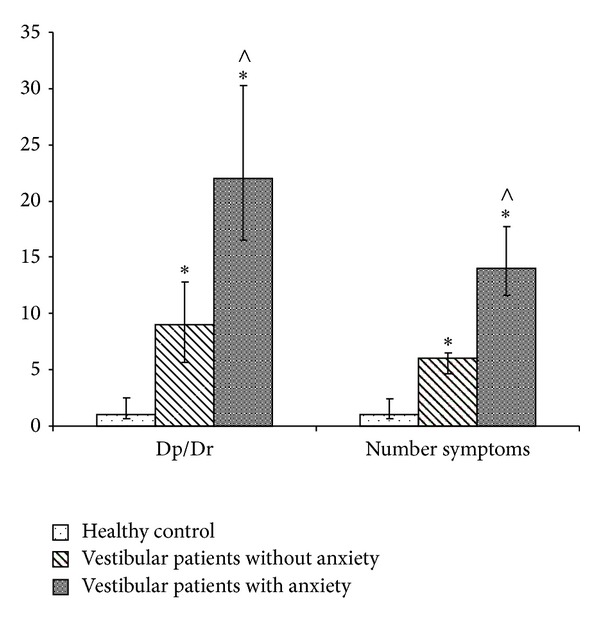
Median values and 95% confidence interval of Dp/Dr total score and number of symptoms for healthy subjects, vestibular patients without anxiety, and vestibular patients with anxiety. *Significance between healthy subjects and vestibular patients, *P* < 0.05 and ^∧^significance between both vestibular patients groups, *P* < 0.05 (Mann-Whitney *U* test and Fisher's exact test).

Healthy subjects reported 0 to 7 symptoms of Dp/Dr (median 1), and the range of the total score was from 0 to 8 (median 1) (Figure 2). The most frequent symptoms (Table 3) were “déjà vu” (25%), “difficulty concentrating” (25%), and “time seems to pass very slowly” (20%). All other symptoms were mentioned by less than 20% of the subjects (Table 3).

Healthy subjects showed a significant, positive correlation only between the Dp/Dr total score and the HADS-A subscale score (Spearman's correlation *r* = 0.444, *P* < 0.05). There were no significant correlations between the Dp/Dr total score and other general characteristics of the subjects, for example, age, gender, healthy habits, marital status, or education (Spearman's test).

### 3.2. Vestibular Patients

The HADS total score of peripheral vestibular patients ranged from 1 to 32 (median 14). The HADS-A sub-score ranged from 2 to 20 (median 8) and the HADS-D sub-score from 1 to 13 (median 6). The number of the Dp/Dr symptoms in vestibular patients ranged from 1 to 26 (median 11), and the Dp/Dr total score ranged from 1 to 78 (average 18). A significant, positive correlation between the Dp/Dr total score and the HADS total score (Spearman's correlation *r* = 0.535, *P* < 0.05) and between the Dp/Dr total score and HADS-A and HADS-D sub-scores (Spearman's correlation *r* = 0.639, *P* < 0.05, Spearman's correlation *r* = 0.377, *P* < 0.05) was observed. Because of the strong correlation between Dp/Dr total score and HADS-A score the group of vestibular patients was divided into two subgroups—vestibular patients without anxiety symptoms with a total score for HADS-A of less than 7 (18 patients) and vestibular patients with anxiety with a total score for HADS-A of more than 7 (24 patients) (Figure 1).

Table 1 presents the differences in the demographic and clinical characteristics between healthy subjects and vestibular patients and between patients with and without anxiety symptoms. A comparison of the two patient groups by age, sex, education level, disease duration, employment, marital status, and healthy habits showed no significant differences. Significant differences (*P* < 0.05, Mann-Whitney *U* test) were found between the two groups for the HADS-A sub-score.

The factors anxiety (*P* < 0.05) and other factors with significant differences (*P* < 0.1) such as age and depression were included in the multiple linear regression analysis as independent variables (Table 4). The findings suggest that Dp/Dr symptoms are significantly associated with anxiety (HADS-A) (adjusted *R* square = 0.579, Durbin-Watson = 2.089 for Dp/Dr total score and adjusted *R* square = 0.552, Durbin-Watson = 2.616 for number of the Dp/Dr symptoms) (Table 4).

The frequency and severity of the Dp/Dr symptoms reported by the vestibular patients were significantly higher on 26 of the 28 items compared to healthy subjects (Table 3). For vestibular patients the number of the Dp/Dr symptoms and Dp/Dr total score were also significantly higher compared to healthy subjects (Fisher's exact test, *P* < 0.05). Apart from “dizziness” (87%) and “feel as if walking on shifting ground” (67%), the most frequent symptoms were “feel “spacy” or “spaced out”” (61%), “vision is dulled” (54%), “feel confused or bewildered” (50%), “difficulty focusing attention” (50%), “feeling of not being in control of self” (48%), and “feel as though your personality is different” (48%). All these symptoms were reported by about 50% of all vestibular patients but were rare in the healthy subjects group.

Discriminant function analysis of severity rating on each item was used to identify the items that could best discriminate anxiety in the vestibular patients. The symptoms “dizziness” and “feel as if walking on shifting ground” are related to vestibular dysfunction. For this reason the subjective score for both groups of vestibular patients for these two items was high. These two items were excluded from the analysis. The combination of four items that best discriminated vestibular patients with anxiety from those without anxiety (Wilk's Lambda of 0.565, *P* < 0.008, Squared Mahalanobis distances 3.07, *P* < 0.003) included “surrounding seems strange and unreal,” “difficulty focusing attention,” “difficulty concentrating,” and “feel confused or bewildered.”

In the group of vestibular patients without anxiety the number of reported Dp/Dr symptoms ranged from 1 to 16 (median 7). The range of the Dp/Dr total score was 1 to 29 (average 9). Only five items were reported in more than 40% of the patients in this group. Apart from “dizziness” (80%) and “feel as if walking on shifting ground” (50%), these were “vision is dulled” (50%), “feel “spacy” or “spaced out”” (45%), and “time seems to pass very slowly” (45%). The Dp/Dr total score was not related to the HADS total score and HADS- A and HADS-D sub-scores or any other characteristics of the patients. No correlation was found between hearing symptoms and Dp/Dr symptoms, neither between vision assessment findings and Dp/Dr symptoms nor other general characteristics of the subjects.

In the group of vestibular patients with anxiety the number of reported Dp/Dr symptoms ranged from 1 to 26 (median 14) and the Dp/Dr total score ranged from 1 to 78 (average 23). In particular 22 of the symptoms were reported by more than 42% of the vestibular patients in this group. Apart from “dizziness” (92%) and “feel as if walking on shifting ground” (81%) some of the most frequent symptoms were “feel “spacy” or “spaced out”” (73%), “feel confused or bewildered” (73%), “time seems to pass very slowly” (69%), “difficulty focusing attention” (65%), “feel as though your personality is different” (62%), “difficulty concentrating” (62%), and “feeling of not being in control of self” (62%). A significant positive correlation between HADS-A sub-score for anxiety and Dp/Dr total score (*r* = 0.388, *P* < 0.05 Spearman correlation) was observed. There was no correlation between HADS-D and Dp/Dr total score. No correlation was found between Dp/Dr symptoms and hearing, vision assessment findings, or other general characteristics of the subjects.

Vestibular patients with anxiety showed a significantly higher total Dp/Dr score and number of symptoms than those without anxiety (Mann-Whitney *U* test, *P* < 0.05). “Déjà vu” was the only symptom reported with a similar frequency in all the groups.

Comparison of the frequency of each of the Dp/Dr symptoms among the groups showed a significant difference for 27 of the 28 items (*P* < 0.05, Mann-Whitney *U* test) between vestibular patients with anxiety and healthy subjects, and for only 9 of the 28 items (*P* < 0.05, Mann-Whitney *U* test) between vestibular patients without anxiety and healthy subjects (Table 3).

A comparison of the frequency of each of the Dp/Dr symptoms between both vestibular patient groups showed significant score differences (Fisher's exact test, *P* < 0.05) for all symptoms except for items 1–5, 9, 10, and 16–18 (Table 3). Two-way ANOVA with factors “duration of disease” (recent and nonrecent) and “anxiety” (without and with anxiety) showed a significant effect only for the factor “anxiety” for both parameters, total Dp/Dr score (*F*(1,41) = 15.22, *P* < 0.001) and number of the symptoms of Dp/Dr (*F*(1,41) = 16.72, *P* < 0.001). The Post hoc analysis revealed significantly higher scores of both parameters only for the group of vestibular patients with anxiety but no significance for the group of vestibular patients without anxiety (Duncan test, *P* < 0.005). The total Dp/Dr scores in the vestibular patients with anxiety and acute vestibular symptoms ranged from 4 to 47 (median 20), and the number of Dp/Dr symptoms from 4 to 26 (median 14), whereas the Dp/Dr total score ranged from 1 to 14 (median 9) and the number of the symptoms of Dp/Dr from 1 to 13 (median 8) for the group of vestibular patients without anxiety and acute vestibular symptoms. The respective values for vestibular patients with nonrecent vestibular symptoms were as follows: the Dp/Dr total scores ranged from 6 to 43 (median 23) for the group with anxiety symptoms and the number of Dp/Dr symptoms ranged from 4 to 25 (median 15) and for the vestibular group without anxiety; the Dp/Dr total scores ranged from 1 to 29 (median 4) and the number of Dp/Dr symptoms from 1 to 16 (median 4).

## 4. Discussion

The present study reveals that anxiety consistently changes Dp/Dr symptoms in vestibular patients. These symptoms are more frequent and more severe in vestibular patients with anxiety compared to those without and they are also qualitatively different. Obviously the factor anxiety is related to the number and score of the symptoms.

In a healthy population the rates of Dp/Dr symptoms are variable and common in daily life [2–4, 28, 29]. The frequency of each of the symptoms of Dp/Dr reported by healthy subjects in this study ranged from 0% to 25%. The frequencies of the symptoms, “déjà vu” (25%) and “difficulty concentrating” (25%), experienced most agree with those reported in previous studies [10, 12, 30]. The results are similar to those found in vestibular patients without anxiety. However, the present study shows that the frequency of these symptoms is considerably lower than in vestibular patients with anxiety. This indicates that anxiety is an important factor in changes of perception.

The results of the comparison of Dp/Dr symptoms in normal subjects and in vestibular patients in general agree with previous findings [10–12, 30]. The symptoms “dizziness,” “feel as if walking on shifting ground,” “feel spacey” or “spaced out,” “surrounding seems strange and unreal,” and “body feels strange/different in some way” were reported consistently more often in vestibular patients (with and without anxiety) than in healthy subjects. These results were similar to those previously reported [10–12, 30]. They can be explained by the vestibular sensory dysfunction, which provokes unreal experiences like vertigo or feelings of sinking on shaking ground. Sensory integration of vestibular information, vision and proprioception fails to occur because the deranged information from the vestibular system does not match with the other sensory inputs and expectations learned by past experience. All these symptoms were evidence for Dp/Dr. We argue that vestibular dysfunction increases Dp/Dr symptomatology by distorting perception [10–12, 30]. The present study revealed that anxiety does not consistently influence these symptoms. In other words, the mechanism of their generation is not essentially related to the anxiety. Therefore, we can assume that anxiety is involved in the generation of some of the Dp/Dr symptoms but not of all.

There is a significant difference in perception depending on the presence or absence of anxiety. Patients with anxiety showed different results in quality and quantity of the Dp/Dr symptoms scored in the questionnaire compared not only to those scored by healthy subjects but also by vestibular patients without anxiety (Table 3). This finding corresponds to the correlation between the HADS-A sub-score for anxiety in vestibular patients with anxiety and the Dp/Dr total score. The inconsistent difference in Dp/Dr symptoms between patients with acute and chronic vestibular disease shows that the factor duration of vestibular pathology is not significantly influencing on the Dp/Dr symptoms.

Symptoms like “feeling as though in a dream,” “feeling of detachment or separation from surroundings,” and “feeling detached or separated from body” have been reported before as evidence for derealization due to the failure of the sensory integration. They occur most frequently and best distinguish patients with vestibular disorders from healthy subjects [10–12, 30]. However, interestingly, we did not find any significant difference in the reports of these symptoms made by healthy subjects and vestibular patients without anxiety (Table 3). Quite the contrary, these symptoms distinguish the vestibular patient group with anxiety from both the vestibular patients without anxiety and from healthy subjects. Another group of symptoms such as “body feels numb,” “numbing of emotions,” “thoughts seem blurred,” “events seem to happen in slow motion,” “your emotions seem disconnected from yourself,” “feel as though in a trance,” “feel confused or bewildered,” and “feel isolated from the world” also indicate a difference between vestibular patients with anxiety and the other two groups studied. The frequency and severity of all these Dp/Dr symptoms apparently are influenced by the presence of anxiety in the vestibular patients.

The balance disorder and complaints of dizziness are associated with elevated levels of anxiety [22–25]. The vestibular symptoms are a frightening experience in general and therefore a major factor leading to the development of an anxiety disorder, especially in those vestibular patients who are predisposed to react adversely to disorientation, whether because of personality traits, behavioral responses, subclinical deficits of perceptual-motor capabilities or cognitive processing, or excessive autonomic nervous system reactivity [31–33]. Therefore, we can probably conclude that the sensory deficit and distorted perception in vestibular patients lead to anxiety and Dp/Dr symptoms to occur. The anxiety in turn facilitates the appearance and the intensity of some of the Dp/Dr (or even alone causes Dp/Dr) symptoms. So obviously, both vestibular disorders and anxiety produce Dp/Dr symptoms. However, part of the vestibular patients are presumably genetically predisposed to anxiety, which in turn generates more frequent and more intensive Dp/Dr symptoms as well as additional Dp/Dr symptoms.

Recently, a cognitive-behavioral model of depersonalization was proposed [21] on the basis of the idea that anxiety and depersonalization are intimately related. The model suggests that if Dp/Dr symptoms are misinterpreted by the patients as indicative of severe mental illness or brain dysfunction, a vicious circle of increasing anxiety and consequently increased Dp/Dr symptoms will result.

Our hypothesis is that dizziness and other vestibular symptoms provoke the experience of derealization, for example, spinning and the shaking of the ground. It probably leads to elevated levels of anxiety in some of the patients, mostly in those who are so predisposed (in their habits), because these experiences are frightening and considered highly life-threatening. The anxiety developed in these patients in turn acts on the vestibular and other integrated systems and increases the number and intensity of already existing vestibular and Dp/Dr symptoms, facilitating the process of sensory disintegration. In this way a vicious circle is created. Dp/Dr and anxiety apparently feed each other, the strangeness and sense of isolation occasioned by depersonalization fuels the anxiety and the depersonalization then intensifies as a defense against this anxiety. We suggest that this background is the major factor causing Dp/Dr symptoms to increase in number and intensity.

Symptoms like “numbing of emotions,” “your emotions seem disconnected from yourself,” and “feel isolated from the world” were reported significantly more often by the vestibular patients with anxiety than by healthy subjects and vestibular patients without anxiety; they reveal the loss of emotional reactivity. People with depersonalization frequently report reduction or loss of emotional responses. Recent functional neuroimaging [34] and psychophysiological [35] studies have found objective evidence of an abnormal response to emotional stimuli, consistent with patient reports of the loss of emotional reactivity. It has been hypothesized that “depersonalization is a hard-wired vestigial response for dealing with extreme anxiety, combining a state of increased alertness with a profound inhibition of the emotional response system.” The proposed mechanism is that the medial prefrontal cortex inhibits the emotional processing of the amygdala and related structures in response to increased anxiety resulting in a dampening of sympathetic output and reduced emotional experiencing that leads to hypervigilance, attentional difficulties, and emptiness of the mind. Sierra and Berrios [36] also suggested that in order to explain how depersonalization can be sensory-modality specific in different patients, the putative disconnection may occur at an earlier stage of emotional processing. To interpret complex and ambiguous inputs the nervous system may use prior knowledge or assumptions, which are constantly adapted by interactive experience with the environment [37]. The role of the limbic system and the amygdala in particular is very important, since affective memory connections to past experience could be an important factor in making new perceptions feel familiar and real [38]. On the other hand, “vestibular dysfunction” is supposed to trigger or cause anxiety syndromes owing to dysfunctional neuronal circuitry exactly in areas such as the hippocampus, amygdala, and infralimbic cortex [39]. Vestibular and visceral information, as well as somatic nociceptive inputs, converge in the parabrachial nucleus, which has reciprocal connections to the central nucleus of the amygdala and the infralimbic and insular cortex and is under the control of higher cortical cognitive regions. We suggest that the emotional hyporeactivity in vestibular patients with anxiety is due to the anxiety. Supposedly the limbic system (insula and amygdala in particular), which is interrelated with the vestibular system, is involved in the generation of Dp/Dr symptoms that reveal an emotional hyporeactivity.

The limitations of this investigation are its cross-sectional design and reliance on self-reports. In the present study we found a correlation between anxiety and Dp/Dr symptoms in vestibular patients. It would be interesting to investigate a relation between those symptoms and the disability caused by the vestibular disease. It was not our objective in this study however, therefore, it would be of interest to be done in a further step.

In conclusion, the present study reveals that anxiety is an essential factor in vestibular patients. It consistently influences the appearance and the intensity of depersonalization and derealization symptoms. This could be of practical benefit in devising a treatment strategy for vestibular patients.

## Figures and Tables

**Table 1:** General characteristics of the subjects and comparisons between groups.

Variables	Healthy subjects *n* = 18	Vestibular patients *n* = 42	*P* value	Vestibular with anxiety *n* = 24	Vestibular without anxiety *n* = 18	P* value
Age (mean ± SD)	41.3 ± 9.5	42 ± 10.6	0.342	44.1 ± 11.1	38.2 ± 13.6	0.074
Gender (female)	72%	83%	0.258	91.7%	72.2%	0.181
Education level (University)	55%	54%	0.59	58%	54%	0.55
Employment status (employed)	90%	72%	0.127	80%	60%	0.174
Marital status (married)	70%	63%	0.322	65%	60%	0.479
Smokers	40%	30%	0.378	35%	25%	0.483
Alcohol (no alcohol)	30%	46%	0.164	38%	55%	0.197
Headache	10%	21%	0.483	25%	17%	0.397
Disease duration (more than 1 year)		50%		54%	44%	0.377
HADS-A subscore (mean ± SD)	2 ± 1.15	10 ± 4.9	0.025*	13.3 ± 4.3	8.1 ± 4.6	0.037*
HADS-D subscore (mean ± SD)	1 ± 1.64	6 ± 3.3	0.017*	6.8 ± 3.5	5.1 ± 2.8	0.087

*P*: difference between healthy subjects and vestibular patients; P*: difference between vestibular patients with and without anxiety; *significant difference—*P* < 0.05 Mann-Whitney *U* test.

**Table 2:** Clinical diagnosis of the vestibular patients participating in the study.

Diagnosis	Vestibular patients *n* = 42	Vestibular patients with anxiety *n* = 24	Vestibular patients without anxiety *n* = 18
Unilateral canal paresis	33	18	14
Vestibular neuritis	30	16	13
Unilateral labyrinthopathy	3	2	1
Bilateral hypofunction/bilateral labyrinthopathy/	3	2	1
BPPV—normal horizontal VOR	6	4	2

BPPV: Benign Paroxysmal Positional Vertigo; VOR: vestibulo-ocular reflex.

**Table 3:** Frequency for each symptom included in the Cox and Swinson Dp/Dr inventory in the three groups participating in the study.

Depersonalization/derealization symptoms	Healthy subjects, *n* = 18	Vestibular patients, *n* = 42	Vestibular patients without anxiety *n* = 18	Vestibular patients with anxiety *n* = 24
(1) Surrounding seems strange and unreal	0%	41%*	30%*	50%*
(2) Time seems to pass very slowly	20%	59%*	45%	69%*
(3) Body feels strange/different in some way	0%	44%*	30%*	54%*
(4) Feel like you've been here before (déjà vu)	25%	41%	30%	50%
(5) Feel as though in a dream	5%	22%	15%	27%*
(6) Body feels numb	5%	28%*	10%	42%^∗●^
(7) Feeling of detachment or separation from surroundings	0%	26%*	5%	42%^∗●^
(8) Numbing of emotions	0%	30%*	15%*	42%^∗●^
(9) People and objects seem far away	10%	35%*	30%	39%*
(10) Feeling detached or separated from body	0%	15%*	5%	23%*
(11) Thoughts seem blurred	5%	41%*	15%	62%^∗●^
(12) Events seem to happen in slow motion	5%	26%*	10%	39%^∗●^
(13) Your emotions seem disconnected from yourself	0%	28%*	5%	46%^∗●^
(14) Feeling of not being in control of self	5%	48%*	30%*	62%^∗●^
(15) People appear strange or unreal	5%	30%*	15%	42%^∗●^
(16) Dizziness	10%	87%*	80%*	92%*
(17) Surroundings appear covered with a haze	10%	33%*	20%	42%*
(18) Vision is dulled	5%	54%*	50%*	58%*
(19) Feel as if walking on shifting ground	10%	67%*	50%*	81%^∗●^
(20) Difficulty understanding what others say to you	0%	30%*	15%*	42%^∗●^
(21) Difficulty focusing attention	5%	50%*	30%*	65%^∗●^
(22) Feel as though in a trance	5%	28%*	15%	39%^∗●^
(23) The distinction between close and distant is blurred	0%	28%*	15%*	39%^∗●^
(24) Difficulty concentrating	25%	50%*	35%	62%^∗●^
(25) Feel as though your personality is different	0%	48%*	30%*	62%^∗●^
(26) Feel confused or bewildered	5%	50%*	20%	73%^∗●^
(27) Feel isolated from the world	0%	34.8%*	10%	54%^∗●^
(28) Feel “spacey” or “spaced out”	0%	61%*	45%*	73%^∗●^

*P < 0.05: significance between healthy subjects and vestibular patients; ^●^ *P* < 0.05: significance between vestibular patients with and without anxiety (Fisher's exact test).

**Table 4:** Multiple linear regression analysis of predictors for Dp/Dr of vestibular patients.

Variables	Multiple linear regression coefficient (*β*)	SE (*β*)	*P* value
Dp/Dr total score			
HADS-A	0.467	0.174	0.01*
HADS-D	0.151	0.175	0.39
Age	−0.037	0.132	0.84
Number of Dp/Dr symptoms			
HADS-A	0.556	0.176	0.003*
HADS-D	0.01	0.178	0.957
Age	−0.034	0.13	0.801

*Statistical significance, *P* < 0.05.
